# *Notes from the Field:* Aircraft Wastewater Surveillance for Early Detection of SARS-CoV-2 Variants — John F. Kennedy International Airport, New York City, August–September 2022

**DOI:** 10.15585/mmwr.mm7208a3

**Published:** 2023-02-24

**Authors:** Robert C. Morfino, Stephen M. Bart, Andrew Franklin, Benjamin H. Rome, Andrew P. Rothstein, Thomas W. S. Aichele, Siyao Lisa Li, Aaron Bivins, Ezra T. Ernst, Cindy R. Friedman

**Affiliations:** ^1^Ginkgo Bioworks, Boston, Massachusetts; ^2^Division of Global Migration and Quarantine, National Center for Emerging and Zoonotic Infectious Diseases, CDC; ^3^Department of Civil & Environmental Engineering, Louisiana State University, Baton Rouge, Louisiana; ^4^XpressCheck, XWELL, New York, New York.

As SARS-CoV-2 testing declines worldwide, surveillance of international travelers for SARS-CoV-2 enables detection of emerging variants and fills gaps in global genomic surveillance (*1*). Because SARS-CoV-2 can be detected in feces and urine of some infected persons (*2*), wastewater surveillance in airports and on aircraft has been proposed by the global public health community^†^ as a low-cost mechanism to monitor SARS-CoV-2 variants entering the United States. Sampling wastewater directly from aircraft can be used to link SARS-CoV-2 lineage data with flight origin countries without active engagement of travelers (*3*).

During August 1–September 9, 2022, the biotech company Ginkgo Bioworks, in collaboration with CDC, evaluated the feasibility of SARS-CoV-2 variant detection in aircraft wastewater from incoming international flights. Aircraft wastewater samples were collected from selected flights from the United Kingdom, Netherlands, and France arriving at John F. Kennedy International Airport in New York City. Wastewater (approximately 0.25 gal [1 L]) was collected from each plane during normal maintenance using a device that attaches to the lavatory service panel port and the lavatory service truck hose.

After concentration with affinity-capture magnetic nanoparticles (*4*), wastewater samples were tested for SARS-CoV-2 by reverse transcription–polymerase chain reaction (RT-PCR).^§^ Samples with cycle thresholds <40 underwent whole genome sequencing using ARTIC (version 4.1; ARTIC Network) primers.^¶^ Multiple lineages within samples were identified using Freyja, a tool for deconvolution of complex samples.** Sequences meeting quality control criteria (e.g., >70% genome coverage)^††^ were assigned to sublineages using Pangolin (version 4.1.3)^§§^ and reported to the airline, public SARS-CoV-2 genomic data repositories, and the CDC National Wastewater Surveillance System. This activity was reviewed by CDC and was conducted consistent with applicable federal law and CDC policy.^¶¶^

During August 1–September 9, 2022, one sample was collected from each of 88 flights (Figure). Sample collection added approximately 3 minutes to normal aircraft maintenance times. Eighty samples were tested for SARS-CoV-2.*** Overall, 65 samples (81%) were positive; the percentage that were positive was similar among the three flight origin countries sampled (Netherlands: 81% [22 of 27]; France: 81% [22 of 27]; and United Kingdom: 81% [21 of 26]). Twenty-seven SARS-CoV-2 genomes were detected in 25 wastewater samples; sequencing quality control criteria were not met for the remaining 40 positive samples. All identified genomes were Omicron sublineages (United Kingdom: 12 BA.5 and one BA.4.6; France: eight BA.5; and Netherlands: five BA.5 and one BA.2.75). In each of 23 samples, single SARS-CoV-2 genomes were identified and assigned to the BA.5 (21), BA.4.6 (one), and BA.2.75 (one) sublineages. In each of two additional samples, two distinct SARS-CoV-2 genomes were identified and assigned to different BA.5 sublineages (Figure). The SARS-CoV-2 genomes identified in aircraft lavatory wastewater were consistent with Western European sequences uploaded to the Global Initiative on Sharing Avian Influenza Data (GISAID) at the time (approximately 90% BA.5).^†††^

**FIGURE:**
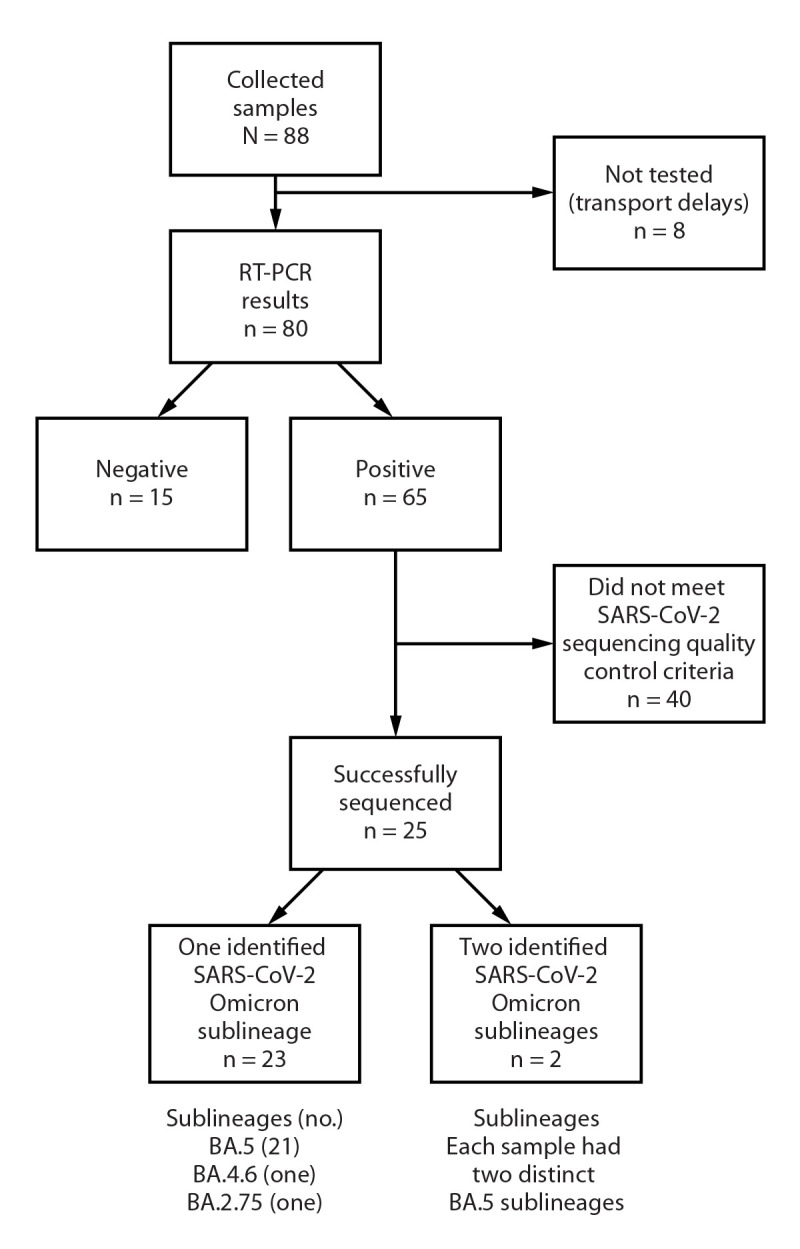
Collection, testing for SARS-CoV-2, and genomic sequencing of aircraft wastewater samples from selected flights from the United Kingdom, Netherlands, and France — John F. Kennedy International Airport, New York City, August–September 2022 **Abbreviation:** RT-PCR = reverse transcription–polymerase chain reaction.

This investigation demonstrated the feasibility of aircraft wastewater surveillance as a low-resource approach compared with individual testing to monitor SARS-CoV-2 variants without direct traveler involvement or disruption to airport operations. Limitations include dependence on lavatory use during the flight, which correlates with flight duration (*5*); inability to distinguish travelers with connecting flight itineraries, which lessens precision when ascertaining variant origin; and potential carryover of residual SARS-CoV-2 RNA between flights yielding viral detections unrelated to travelers on the flight. Stringent genome coverage thresholds might reduce the likelihood of carryover variant identification on subsequent flights.

In addition to routinely monitoring variants entering the United States, this modality can be surged based on global public health needs (e.g., outbreaks or mass gatherings in settings with limited SARS-CoV-2 variant surveillance). In combination with traveler-based surveillance (*1*), aircraft wastewater monitoring can provide a complementary early warning system for the detection of SARS-CoV-2 variants and other pathogens of public health concern.

## Footnotes

†
https://wastewater-observatory.jrc.ec.europa.eu/static/pdf/Sampling%20Aircrafts_FINAL_Version%209%20Jan%202023.pdf

§ Preliminary studies indicated no inhibition of RT-PCR reagents by lavatory fluid.

¶
https://github.com/artic-network/artic-ncov2019/tree/master/primer_schemes/nCoV-2019

**
https://github.com/andersen-lab/Freyja

†† Coverage determines whether variant assignment can be made with a certain degree of confidence. The coverage percentage is the proportion of the reference genome aligning with the consensus genome generated during sequencing.

§§
https://cov-lineages.org/resources/pangolin.html

¶¶ 45 C.F.R. part 46.102(l)(2), 21 C.F.R. part 56; 42 U.S.C. Sect. 241(d); 5 U.S.C. Sect. 552a; 44 U.S.C. Sect. 3501 et seq.

*** Eight samples were not tested because of transport delays.

†††
https://covariants.org/
